# Physico-Mechanical Properties and Microstructure of Polymer Concrete with Recycled Glass Aggregate

**DOI:** 10.3390/ma11071213

**Published:** 2018-07-14

**Authors:** Bartosz Zegardło, Maciej Szeląg, Paweł Ogrodnik, Antoni Bombik

**Affiliations:** 1 Faculty of Natural Sciences, Siedlce University of Natural Sciences and Humanities, 2 Stanisława Konarskiego Str., 08-110 Siedlce, Poland; bart.z@wp.pl (B.Z.); antoni.bombik@uph.edu.pl (A.B.); 2 Faculty of Civil Engineering and Architecture, Lublin University of Technology, 40 Nadbystrzycka Str., 20-618 Lublin, Poland; 3 Faculty of Fire Safety Engineering, The Main School of Fire Service, 52/54 Słowackiego Str., 01-629 Warsaw, Poland; pogrodnik@sgsp.edu.pl

**Keywords:** polymer concrete, glass waste, glass recycling, lighting elements

## Abstract

The paper presents an analysis of the possibility of using glass waste from worn out lighting materials as an aggregate for a polymer concrete. Glass waste was obtained from the company utilizing glass lighting elements, which was then subjected to crushing. The aggregate obtained was subjected to the tests of basic features, which were compared with aggregates that are traditionally applied to concretes. The next stage of the research program was the production of a polymer concrete that contained glass aggregate. Several types of mixtures were prepared in which glass waste was combined in various proportions with traditional sand–gravel aggregate. As a part of the research, the basic physical and mechanical characteristics of polymer concretes were determined. The microstructure of composites was also analyzed using a scanning electron microscope. The results of the research have shown that the aggregate obtained from glass waste can be successfully used for the production of a polymer concrete. The most beneficial physico-mechanical properties were obtained for a composite in which glass waste was used as a 50% substitute for traditional aggregate.

## 1. Introduction

With the invention of electric current and the general electrification of the planet, the lighting industry developed. Although the current sources of light are energy-efficient and have a very long service life, they eventually wear out and have to be replaced with new ones. Worn out lighting elements are then industrial waste, which is difficult to dispose [1]. 

The main components of lighting elements are glass and metal elements that are closely connected to each other. Regarding the re-use of the materials, this is a problem, because the direct recipients of the waste cannot be manufacturers of lighting elements, which is the case in, e.g., the typically steel or glass industry. The waste is delivered to intermediary companies that adapt the material for re-use. These processes are often technologically complex, which economically affects the whole issue [2]. To produce perfectly transparent lighting glass, the waste must not contain any impurities before it can be remelted. In addition, processes such as collection, segregation, and transport increase the cost of recycling [3].

In connection with the above causes, other methods of recycling glass waste are sought in which the adjustment of the waste to new production does not have such high requirements. An industry that is often mentioned in this place is the production of construction products. The use of glass for the production of, e.g., concrete composites could potentially bring some benefits [4,5,6]. Entrepreneurs producing composites could obtain material for the production, which would be relatively cheap after separating steel elements. In addition, the extraction of traditional aggregates would be limited, because they would be replaced by recycled material. 

The use of glass for concretes is a known process, and depending on the form in which it is used, several directions of such activity can be distinguished. The first is the use of glass fibers [7,8,9,10]. Glass fibers that are produced specially for this purpose improve the strength parameters of composites, and make them less sensitive to atmospheric factors such as moisture, insolation, or frost. However, such activity requires glass melting, and cannot be carried out locally.

Another trend in the use of glass in composites is the use of glass dust. Research [11,12,13] has shown that glass dust can significantly increase the strength parameters of concretes. However, this is achieved with a very high degree of glass dust fragmentation, which is an expensive process. 

The relatively newest trend, and at the same time the most economically justified, is the use of inorganic waste in the form of coarse aggregate [14,15,16,17,18,19,20]. Research work conducted on the basis of glass waste obtained, e.g., from LCD monitors prove that the parameters of concretes (tensile and compressive strength, water absorption, resistance to low and high temperatures) with such an addition do not differ from those obtained using traditional aggregates. For the preparation of glass aggregate, crushers can be used exactly as they are used on concrete plants. In addition, the production cycle of composites is the same as that used for traditional aggregates. An additional advantage of this solution is the possibility of using glass waste locally, which eliminates the transport costs.

Other studies [21,22,23] have shown that cement-based materials containing glass aggregates are subjected to the alkali silica reaction, which is not beneficial in the aspect of durability of the material. However, this problem does not apply to concretes containing only a polymer matrix as a binder.

The paper describes the results of studies related to the use of waste glass from exploited lighting elements as filler for the polymer concretes. The waste material was obtained from a company that utilizes worn out lighting elements. Glass from discarded light bulbs was subjected to grinding, and aggregate was made of it. The aggregate produced was subjected to the examination of the basic technical features to determine its properties. Six different polymer concretes were produced that contained crushed waste glass and conventional sand–gravel aggregate in varying proportions by weight. The composites obtained underwent basic physical and mechanical tests; the microstructure of the concrete was also analyzed.

## 2. Materials and Methodology

### 2.1. Polymer Matrix

Previous attempts [24,25,26] to produce polymer concretes using recycled materials have shown that the most beneficial effects, both aesthetic and strength, are obtained using unsaturated orthophtalic polyester resins. Their main advantage is the transparent color, which does not change after hardening. An additional advantage of these resins is the possibility of mixing them with transparent dyes, which give it many-colored shades, while the resin remains transparent. For the tests, a colorless, transparent, unsaturated orthophtalic polyester resin was used: the Crystal Clear Synolite 1881 (Wollaston, Northamptonshire, UK) by Scott Bader. The basic resin parameters are shown in Table 1.

The initiator—the Butanox M-60 (Arnhem, Netherlands)—was used to cure the resin. It was used according to the manufacturer’s recommendation in an amount of 3% relative to the weight of the resin used. The basic properties of the initiator are shown in Table 2. In order to give the composites aesthetic qualities, transparent dyes for resins of different colors were used.

### 2.2. Traditional and Glass Waste Aggregate

**Figure 1:**
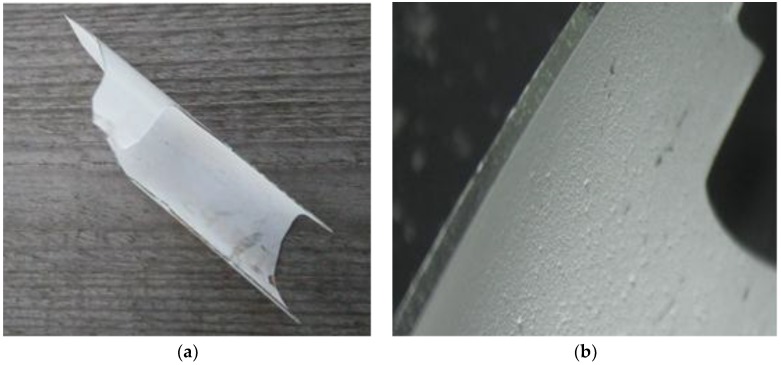
Wastes from lighting glass: (**a**) part of fluorescent lamp; (**b**) view of glass grain under an optical microscope.

The glass waste was obtained from a company dealing with the utilization of electric waste. Worn out light bulbs and fluorescent lamps were devoid of any non-glass tooling. During this process, their original shape was damaged. The material obtained was taken from the heap and transported to the laboratory. The recycled glass aggregate (RGA), which is shown in Figure 1, was made by grinding the glass waste in a steel mortar. The grinding process was carried out until all of the grains were screened through a sieve with an 8-mm mesh.

In the case of obtaining a glass waste aggregate, the processing procedure is very important, because fluorescent lamps contain traces of mercury that could be harmful to health. The glass waste obtained from a recycling company was free from chemicals that adversely affect health. The process of utilizing fluorescent lamps was carried out under negative pressure, so that mercury vapor could not get into the atmosphere. First, the used fluorescent lamps were put in a feeder, which was tightly closed, and then its content reached the chamber, which was heated above 200 °C. As a result of heating, the evaporation of mercury followed, while the glass and aluminum tips remained in the chamber at the bottom. After filling the chamber with glass and aluminum, its contents were directed to the separator. Floating mercury vapor was directed to the next chamber, where the temperature was –75 °C, which caused the mercury to shift from a gaseous to a liquid state. The separation of mercury meant that it could be re-used, and the final product in the form of the glass waste was free of compounds posing a health risk.

**Figure 2:**
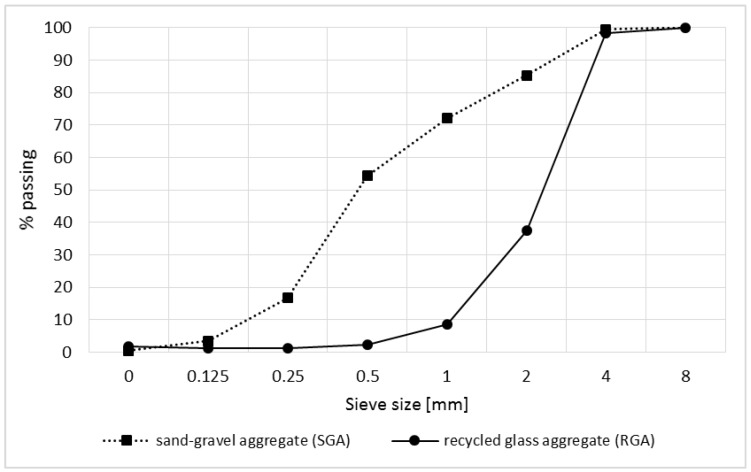
Particle size distribution of the aggregates used.

In the study, the traditional aggregate in the form of a suitably composed mixture of sand and gravel (SGA) was also used. The particle size distribution of aggregates used in the study is shown in Figure 2.

### 2.3. Aggregates Parameters Testing

Basic features [27,28] were defined for RGA, which are examined for aggregates traditionally used for cementitious composites. For comparison purposes, the same tests were performed for traditional aggregate, SGA. The specific density was tested according to EN 1097-7 [29]. Apparent density and water absorption were determined according to EN 1097-6 [30]. The 0–8 mm fraction and 4–8 mm fraction were separated by the sieve method on which the test of crushing strength was conducted in accordance with PN-B-06714-40 [31]. The test was performed in a hydraulic press using a special vessel with a piston for crushing the aggregate. The crushing degree was set as a percentage share of the grains, which after crushing passed through a sieve of 1-mm mesh.

In order to assess the shape of RGA grains, a comparative method was used [27,32]. The test was carried out on representative samples taken from 0–2 mm, 2–4 mm, and 4–8 mm grain sizes. The length, width, and thickness of the grains were measured using an optical microscope. The length and width were at least three times greater than the thickness; therefore, the glass aggregate grains were classified as flat grains with a smooth surface.

### 2.4. Mixtures of Polymer Concretes

The composition of the concrete was designed by the iterative method, in which wet ingredients were added to the combined dry ingredients in increasing quantities. This process was repeated until the free spaces between the grains were filled with the binder. The composition of the resin concrete with RGA that was obtained in this way is shown in Table 3.

In order to determine the effect of the waste glass aggregate content on selected physico-mechanical features of polymer concretes, several types of mixtures were made, in which the recycled aggregate was a substitute (by weight) of traditional aggregate. Six mixtures designated as CG-0, CG-10, CG-30, CG-50, CG-70, and CG-100 were performed; the number in the designation indicates the percentage content of the RGA.

### 2.5. Testing the Properties of the Polymer Concretes

The polymer composites have been subjected to basic technical tests for concrete used in building constructions. The bulk density, water absorption, water permeability, tensile strength in a three-point bending, and cubic compressive strength were tested.

The bulk density was tested on rectangular samples with dimensions of 40 × 40 × 160 mm^3^. The test was carried out in accordance with EN 12390-7 [33]. The results obtained are the arithmetic mean of six samples.

The water absorption was tested on identical samples as bulk density, in accordance with the rules given by Neville [27]. The samples were immersed in water and allowed to reach the maximum mass. The water absorption was calculated as the percentage ratio of the amount of water that the composite was able to absorb to the mass of dry material. Six samples of each of the composites were tested.

Six samples from each recipe with dimensions 100 × 100 × 100 mm^3^ were prepared for the water resistance test. The parameter was determined by applying a one-sided hydrostatic pressure of 0.2 MPa for 24 h, and raising the water pressure by 0.2 MPa every 24 h until leakage symptoms appeared. The measure of water resistance was the maximum water pressure that did not cause leakage. The test was carried out in accordance with EN 12390-8 [34].

The tensile strength was determined in accordance with EN 12390-5 [35] in a three-point bending scheme. As the largest diameter of the aggregate used was 8 mm, the test could be carried out on 40 × 40 × 160 mm^3^ samples. The results are presented in the form of an arithmetic mean of six samples. 

The cubic compressive strength test was carried out in accordance with EN 12390-3 [36]. Samples measuring 40 × 40 × 40 mm^3^ were made after fracturing samples were prepared for tensile strength testing. The results obtained are the arithmetic mean of 12 samples.

An analysis of the local microstructure of the polymer concrete was carried out based on images obtained from SEM. The samples for the analysis were broken off from the inner part of the sample. To obtain a conductive layer, the samples were sputtered with carbon. The photos were taken in a high vacuum mode.

The resin used achieved a constant non-increasing strength after 16 h. Despite this, all of the tests mentioned above were carried out after 28 days from the day the samples were made. It was in accordance with the recommendations as for cement concrete.

## 3. Results and Discussion

### 3.1. Evaluation of the Properties of the Recycled Glass Aggregate

Table 4 summarizes the results of the basic parameters of the RGA and SGA. A comparison of the aggregate features obtained from glass waste shows that it has both more and less beneficial properties compared with traditional aggregate. The advantageous feature is a much higher compressive strength (almost 20 times) and almost zero water absorbability. The reason for this is a very tight RGA structure (which in the further part of the article is shown in the SEM images), and thus very low porosity. The negative feature of the glass grains is their flat shape and the high value of the crushing degree. The RGA shape may cause tendentious arranging grains in the mix, which with a high content of the recycled aggregate, may result in the deterioration of the mechanical properties of the polymer concrete.

A quantitative description of the shape of the aggregate was obtained on the basis of measurements of the aspect ratios, i.e., a/b (thickness/width) and a/c (thickness/length). The a/b ratio was equal to 0.34 with a standard deviation of 0.19, while the a/c ratio was equal to 0.20, with a standard deviation of 0.05.

### 3.2. Physical Characteristics of Polymer Concrete with RGA

The results of bulk density, water absorption, and water resistance tests are presented in Table 5. The bulk density of polymer concrete increased with the increase of RGA content in the mixture. The lowest bulk density was obtained for the composite, which was used in 100% traditional aggregate. It was 8.8% lower than the highest value, which was for polymer concrete with 100% RGA content. As shown previously, both types of the aggregates have very similar specific density; however, due to the higher porosity of SGA, the difference is in the bulk density. The pores contained in the volume of the traditional aggregate made the composites lighter than those in which the RGA content was greater.

In the case of water absorption, the inverse relationship was observed. With the increase of the RGA content in concrete, its water absorption decreased, reaching a minimum value of 1.22% for CG-100. The polymer concrete, in which only SGA (CG-0) was used, achieved water absorption by 27% higher. Cementitious concretes, depending on the composition, achieve water absorption that was two to four times larger [18,38,39] than the values obtained for the polymer concretes. Such low absorbability is caused by the low absorbability of the binder itself, which was in this case the polyester resin. The polymer matrix is very tight, and to a large extent, it limits the penetration of water into the composite. In contrast, the differences in water absorption between the concretes tested result from the absorbability of the aggregates used; RGA has a very tight, little porous structure, which is practically not absorbing water. This was also confirmed by other researchers [40].

All of the concretes tested obtained a water resistance class higher than W6. A hydrostatic pressure of 6 MPa did not cause any leakage. The highly waterproof polymer matrix is mainly responsible for the high water resistance.

**Figure 3:**
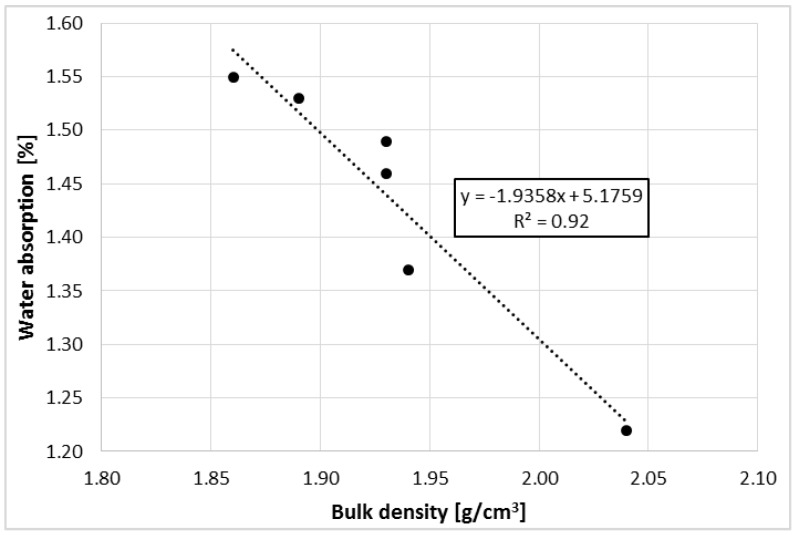
Water Absorption of the Polymer Concretes with RGA as a Function of Bulk Density.

Figure 3 shows the relationship between the water absorption of polymer concretes and their bulk density. The Pearson’s correlation coefficient between these parameters has a very high value that is equal to −0.96. In this case, as the bulk density increases, the absorbability of the concretes decreases, but as mentioned earlier, it is related to the increasing content of RGA in the mix. The proposed linear regression model between these parameters covers empirical data in 92%, which indicates very good model matching. An attempt to estimate the water absorption of the polymer concretes tested based on the bulk density would have a very small error.

### 3.3. Tensile Strength of the Polymer Composites with RGA

**Figure 4:**
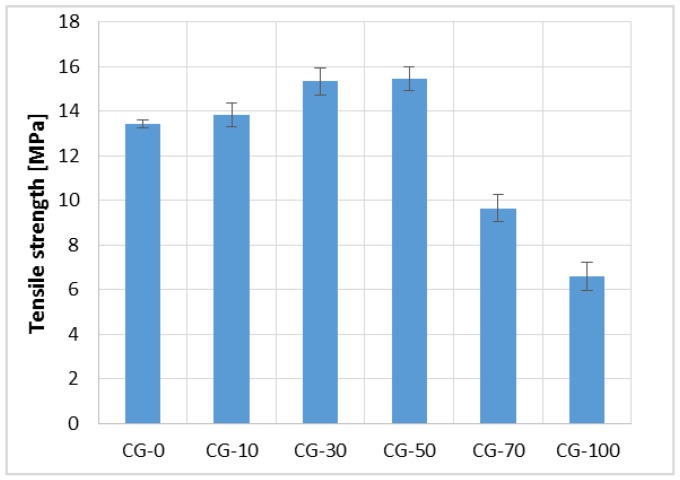
Tensile Strength of the Polymer Concretes (in the form of error bars, the standard deviation is shown).

The results of the bending tensile strength test are shown in Figure 4. It was noted that as the RGA content increases to 50%, the strength increases. However, for higher RGA contents in the mix, this parameter has deteriorated. The composite with 50% RGA content obtained the highest tensile strength value of 15.44 MPa, which was 14% higher compared with the reference composite, CG-0. In contrast, CG-100 achieved the strength value equal to 6.61 MPa, which accounted for 49% and 46% of the values obtained for CG-0 and CG-50, respectively.

While observing the fractures of the samples after the test, it was noticed that SGA grains, which have an oval shape, were largely debonded from the polymer matrix. However, in the case of RGA grains, two mechanisms of destruction were noticed; in the first case, the RGA grains were pulled out of the polymer matrix (due to the smooth surface of the grains, i.e., low adhesion to the matrix); in the second case, when the RGA grains were surrounded by a binder on each side, their adhesion to the matrix was greater, and the grain itself broke due to its brittleness.

**Figure 5:**
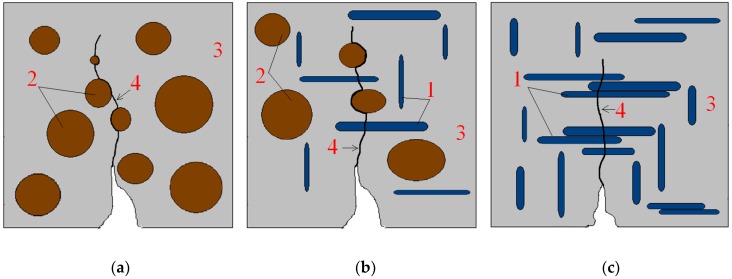
Scheme of Bending Destruction of Polymer Concrete with Different RGA and SGA contents: (**a**) 0% RGA, 100% SGA—CG-0; (**b**) 50% RGA, 50% SGA—CG-50; (**c**) 100% RGA, 0% SGA—CG-100; 1—RGA grain; 2—SGA grain; 3—polymer matrix; 4—zone of destruction.

In the CG-10, CG-30, and CG-50 composites, the RGA grains were evenly distributed in the polymer matrix and surrounded on each side with the binder. Thus, the adhesion of the RGA grains to the matrix was high, and the grains being arranged perpendicular to the direction of the bending force could be a form of micro-reinforcement, which probably caused the increase of tensile strength. Grains of glass prevented the sample from being destroyed until their local tensile strength was exceeded. However, in the CG-70 and CG-100 composites, the RGA grains were subjected to excessive agglomeration, and since they were adhering to each other, it was not possible to fully surround them with the resin. In this case, due to the low adhesion to the matrix, the RGA grains were pulled out of the resin or broken, which resulted in a reduction in the tensile strength. The proposed mechanism of destruction has been schematically depicted in Figure 5.

### 3.4. Cubic Compressive Strength of the Polymer Concretes with RGA

**Figure 6:**
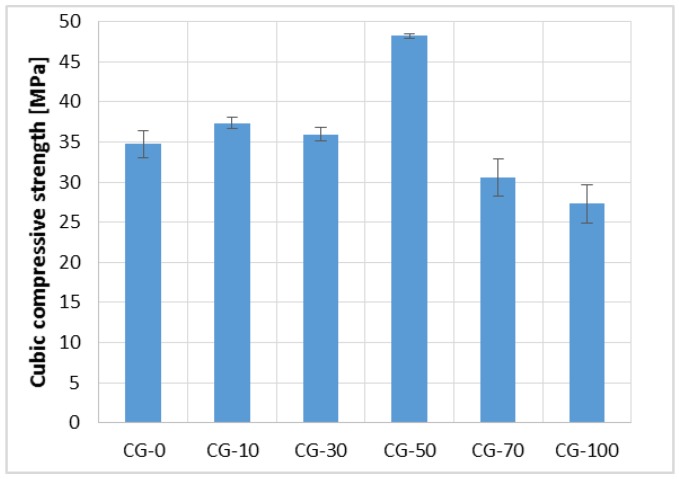
Cubic Compressive Strength of the Polymer Concretes (in the form of error bars, the standard deviation is shown).

The results of the cubic compressive strength test are shown in Figure 6. Similarly as in the case of the tensile strength, an increase in the parameter was observed with an increase in RGA content in the mixture from 0% to 50%. The cubic compressive strength of CG-50 was equal to 48.90 MPa, which was 38% higher than CG-0, and 43% higher than the composite, in which only RGA was used (CG-100). The CG-100 achieved a compressive strength equal to 27.31 MPa, which was 21% lower than the reference concrete, CG-0.

**Figure 7:**
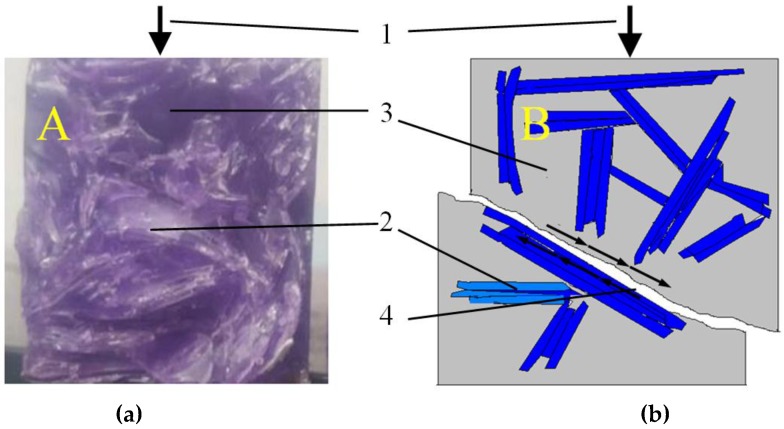
Destruction of the CG-100 Sample Due to Compressive Stress: (**a**) Sample Fracture; (**b**) Scheme of Destruction Due to the Shear at the Interface of the RGA Grain–Polymer matrix; 1—compressive force direction; 2—SGA grain; 3—polymer matrix; 4—zone of destruction.

At the moment when the RGA grains are still relatively dispersed and well surrounded by a polymer matrix (as was in the case with RGA content from 10% to 50%), the glass aggregate may be a micro-reinforcement that bridges the micro-cracks arising from the compressive stress in the resin matrix. This phenomenon may be the reason for the increased compressive strength of CG-10, CG-30, and CG-50 concretes in reference to CG-0 concrete. However, during the examination of CG-70 and CG-100 composites, in which strength was reduced, it was noticed that shearing very often caused the destruction of the sample. The analysis of the fractures of the samples indicated that at the RGA contents of 70% and 100%, during the formation of the composite, the glass aggregate grains were often arranged parallel to each other, adhering to each other by planes. Depending on the orientation of the RGA grains with respect to the compressive force direction, shear stress appeared in the areas of the glass aggregate, which caused the aggregate grains to slip relative to each other, as well as to the polymer matrix. The result was the destruction of the material, with an ever-lower value of the compressive load. Figure 7 shows the destruction zone of the CG-100 sample with visible flat RGA grains adjacent to each other; the process of destruction of the polymer composite with RGA content exceeding the optimum level (>50%) was also schematically shown.

The compressive strength, according to the standard recommendations, can also be tested on elongated samples, e.g., in the shape of a cylinder. The destruction of cubic samples in the compression test is mainly caused by shear-induced stress, while in the case of elongated samples, the trajectory of stress takes a near-vertical direction. In traditional concrete, the material’s uniaxial compressive strength (measured on elongated samples) is known to be about 20% higher than in the case of cubic compressive strength. This is due to the change in the failure mechanism depending on the geometry of the sample. As mentioned above, along with the increase of RGA content in the composite (mainly CG-70 and CG-100), the destruction of specimens was mainly caused by shearing; the RGA grains were arranged parallel to each other. It should be remembered that the increase factor (difference in the compressive strength between cubic and elongated samples) may be completely different, or may not occur at all in the case of the composite tested. With the increasing content of flat RGA grains in the material, the shear failure may begin to dominate during the uniaxial compressive strength test. The result may be lower strength values on elongated samples compared with cubic ones. In addition, there is a probability that for the uniaxial compressive strength test, the highest value of the parameter will be obtained for a composite with different RGA content. However, confirmation of this thesis requires additional tests that the authors plan to perform in their further research.

### 3.5. Relationship Between Compressive and Tensile Strength

**Figure 8:**
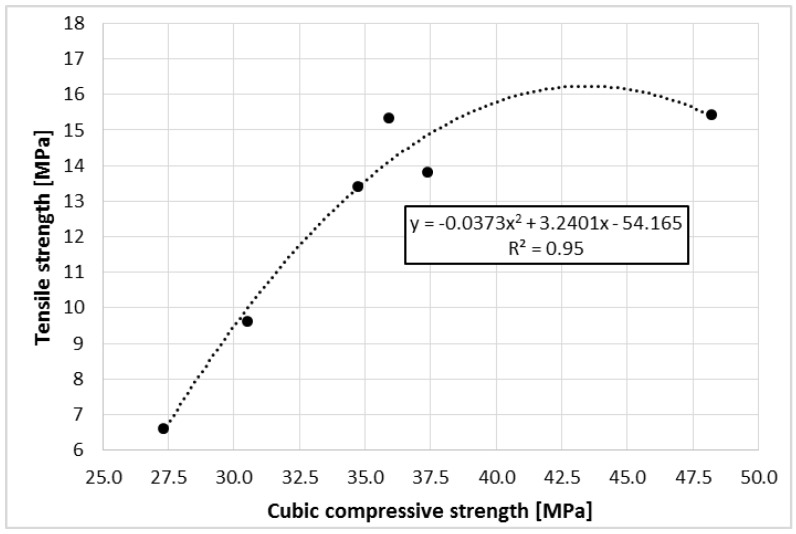
Tensile Strength of the Polymer Concretes with RGA as a Function of Cubic Compressive Strength.

Figure 8 shows the relationship between tensile strength as a function of compressive strength of the polymer concrete containing RGA. The correlation coefficient between these parameters assumes a high value that is equal to 0.81. The dependence of strength parameters does not take a strictly linear relationship, which results from the change of the material destruction mechanism due to the occurrence of a higher RGA content at the expense of SGA. To describe the relationship between strength parameters, a quadratic curve was proposed. The value of the determination coefficient R^2^ equal to 0.95 indicates that the proposed model is very well suited to the empirical data.

In addition, the ratio of tensile strength to cubic compressive strength for the polymer concretes takes very high, advantageous values ranging from 0.242 (CG-100) to 0.427 (CG-30). For comparison, this relation for concretes based on cement binder is much lower, and for conventional cementitious concretes, it ranges between 0.070 and 0.130 [27,41,42]. Very high tensile strength in relation to the cubic compressive strength that was achieved for the polymer concretes allows for the manufacture of long flat elements that would be loaded perpendicular to their axis, without the use of steel reinforcement, which is necessary for traditional cementitious concrete.

### 3.6. Analysis of the Local Microstructure

**Figure 9:**
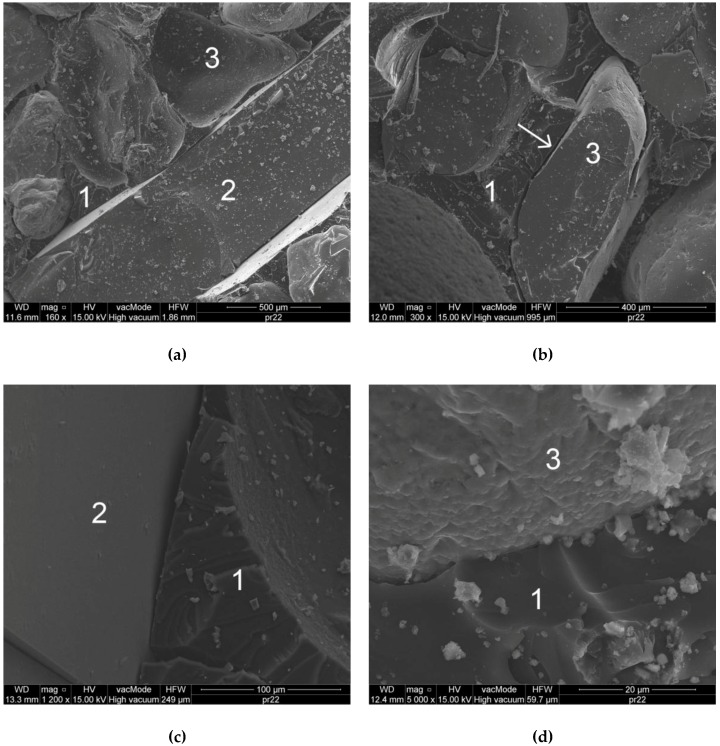
SEM photos of the CG-50 Polymer Concrete Microstructure: (**a**) Sand and Gravel Aggregate (SGA) and RGA Grains Surrounded by a Polymer Matrix; (**b**) SGA Grain Detached from the Matrix; (**c**) Contact of RGA with the Resin Matrix; (**d**) Contact of SGA with the Resin Matrix; 1—polymer matrix; 2—RGA grain; 3—SGA grain.

An analysis of images obtained from SEM allowed for the analysis of material morphology. Figure 9a shows a flat RGA grain with smaller SGA grains. Grains are surrounded on each side with a polymer matrix, which indicates the good mixing of ingredients at the stage of forming the sample. Figure 9b shows the grains of traditional aggregate surrounded by a resin matrix. It was observed that a part of grains are clearly debonded from the matrix, which adversely affects the mechanical characteristics of the polymer concrete. In such places, there is no structural bonding of the aggregate grain with the matrix, which explains the mechanism of destruction that was observed during the tensile strength test (the destruction zone ran through the contact between the SGA grain and the polymer matrix, not through the grain).

Figure 9c shows the RGA grain contact with the resin matrix. It has been confirmed that the surface of the glass is very smooth and tight. The binder can only touch the aggregate without creating any structural bindings. The adherence of the RGA grain to the matrix is then the resultant of the frictional force occurring in the contact. In addition, due to the flat RGA shape, the contact surface is relatively large, which positively affects the adhesion to the matrix. The key aspect in this case is that the grain on each side should be surrounded by resin, which, as demonstrated earlier, is difficult to achieve in the case of RGA content exceeding the optimum level. In the case when the RGA content in the mixture exceeded 50%, the grains tended to agglomerate, and were very often arranged to be in parallel with each other in contact with their planes. Due to the lack of resin between these grains, their mutual adhesion to each other was almost zero, which resulted in the faster destruction of the material with an increase in RGA content above 50%.

Figure 9d shows the SGA grain contact with the polymer matrix. The surface of the grain is rougher than RGA; however, the resultant adhesion of the matrix to the grain is less due to the oval shape of the grain. As in the case of RGA, there was no structural matrix bonding with the SGA grain. The binder reaches the grain without creating a so-called interfacial transition zone (ITZ), as it is in the case of cementitious concrete. This is also because the polymer matrix is very homogeneous in structure, since it consists only of the sequences of the repeating polymer chains. In contrast, cement matrix is very chemically diverse; in a hardened cement paste a number of independent morphological forms can be distinguished, e.g., CSH phase, Portlandite, gypsum, ettringite, etc.

In the high magnification pictures (Figure 9c,d), it is also seen that the polymer matrix, apart from being structurally uniform, is also very tight. The local porosity is practically non-existent, and the internal cohesion of the polymer causes the composite to have a very high tensile strength in relation to compressive strength.

### 3.7. Aesthetic Values of the Polymer Concrete with RGA

**Figure 10:**
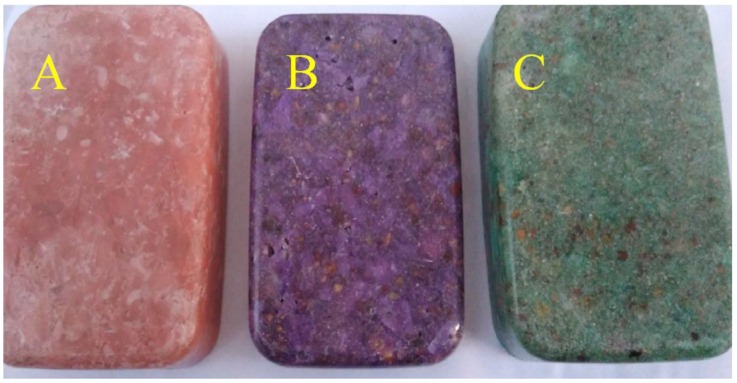
Aesthetics of the Polymer Concretes with Different RGA Content: (**a**) CG-100; (**b**) CG-50; (**c**) CG-30.

Samples of CG-100, CG-50, and CG-30 in different colors are shown in Figure 10. Samples were easy to demold, because they were made in plastic molds. Transparent dyes gave them high aesthetic values. Thanks to the transparency of the polymer matrix, aggregate grains of both SGA and RGA embedded in the binder are visible. Bearing in mind the physico-mechanical properties obtained of the polymer concrete with the addition of RGA, this material can be recommended for elements such as window sills, kitchen countertops, and structural components from exposed composites. In addition, the use of waste material such as recycled glass aggregate is in line with the latest trends in building materials technology, and it is also in line with the principles of sustainable development.

## 4. Conclusions

The article explored the possibility of using glass waste as an aggregate for the production of polymer concretes. On the basis of the analyzed and interpreted research results, final conclusions were formulated:

1. Aggregate made from worn out lighting elements has both beneficial and unfavorable features compared to traditional aggregates used for concretes; the advantage is very high compressive strength and almost zero water absorption; the disadvantage is a high degree of crushing and flat grain shape.
2. The higher the content of recycled glass aggregate in the volume of the polymer matrix, the greater the bulk density and lower water absorption of the polymer concrete.
3. The highest tensile and cubic compressive strength values were obtained by a composite in which 50% of the aggregate consisted of glass aggregate; these values were higher by respectively 14% and 38% than the values obtained for the reference concrete (only with sand–gravel aggregate).
4. In composites in which the amount of waste aggregate was up to 50%, it was noticed that the glass grains were well surrounded by a binder on each side; in this case, the adhesion of the grains to the matrix was high, and the grains that were arranged perpendicular to the direction of the destructive force could be a form of micro-reinforcement that resulted in increased tensile strength.
5. In concretes in which the amount of waste aggregate exceeded 50%, it was noticed that the glass grains tended to agglomerate while at the same time touching their planes; in this case, the surrounding of the grains with the binder was incomplete, so their adhesion to the matrix was low, which resulted in a reduction in both tensile and compressive strength.
6. The zone of destruction in the vicinity of the grains of the traditional aggregate ran through the grain–matrix interface (debonding); in the case of flat grains of glass aggregate, destruction was most often caused by grain breakage, which was anchored in the matrix.
7. In cases of agglomeration of flat grains in a polymer matrix, the destruction occurred due to the shear along adjoining grains.
8. Analysis of the microstructure indicated that the adhesion of glass aggregate grains to the matrix is most likely a resultant of the friction force between the grain surface and the binder; due to the smooth surface of the glass grain, there was no structural bonding of the matrix to the grain.
9. Favorable technical features and interesting aesthetics of the polymer composites with waste glass aggregate mean that the material can be successfully used for the production of such elements as: window sills, kitchen countertops, and structural components from exposed composites.

## Figures and Tables

**Table 1:** Properties of the Crystal Clear Synolite Resin (information from the product’s card).

Parameter	Value
Brookfield’s viscosity at 23 °C	1200 mPa∙s
Specific weight	1.04 g/cm^3^
Time of maintaining the plastic state	20 min
Time of demolding	16 h
Shore’s hardness	80
Breaking strength	17.2 N/mm^2^
Modulus of elasticity	504 N/mm^2^
Elongation at break	10%
Flexural strength	73.35 N/mm^2^
Bending modulus	1377 N/mm^2^
Compressive strength	43.98 N/mm^2^
Shrinkage	0.1
Heat resistance	50 °C
The refractive index at 20 °C	1.49962

**Table 2:** Properties of the Butanox M-60 Hardener (information from the product’s card).

Parameter	Value
Density at 20 °C	1170 kg/m^3^
Viscosity at 20 °C	25 mPa∙s
Temperature of self-ignition	220
Form	transparent liquid
Solubility in water	insoluble
Solubility in flatanes	soluble

**Table 3:** Composition of the Polymer Concrete Mix: CG-100.

Ingredient	Amount [kg/m^3^]	Density [kg/dm^3^]	Volume [dm^3^]	% by Weight
Polyester resin	369.97	1.1	336.34	17.65
Hardener	11.10	1.17	9.49	0.53
Color pigment	5.55	1.29	4.30	0.26
Recycled glass aggregate (RGA)	1709.26	2.63	649.91	81.55

**Table 4:** Basic Properties of the Aggregates Used.

Property	Unit	Traditional Aggregate—SGA	Recycled Aggregate—RGA
Specific density	g/cm^3^	2.65	2.63
Bulk density	g/cm^3^	2.20	2.62
Compressive strength [37]	MPa	33	650
Modulus of elasticity [37]	10^2^ MPa	330	700
Water absorption	%	2.1	0.1
Crushing degree	%	8.3	34.5

**Table 5:** Physical Properties of the Polymer Concrete with RGA.

Property	A Type of Polymer Concrete					
	CG-0	CG-10	CG-30	CG-50	CG-70	CG-100
Bulk density [g/cm^3^]	1.86	1.89	1.93	1.93	1.94	2.04
Water adsorption [%]	1.55	1.53	1.49	1.46	1.37	1.22
Water resistance	above W6					
